# 

**Published:** 1892-05-21

**Authors:** 


					Nr. nnp 1 ?? v 1-11 vua
f'i? Vol. XII.?May 21st, 1892.
General Post Office as a Newspaper for
81011 in the United Kingdom and Abroad.!
No. 295; Vol. XII.] Saturday,
May 21st, 1892.
[Twopence.

				

